# Reconciling Differences Between Podiatric and Orthopaedic Surgeons in the United Kingdom: The Memorandum of Understanding and Its Implications for the Future of Podiatric Surgery

**DOI:** 10.1002/jfa2.70048

**Published:** 2025-04-29

**Authors:** Ewan Kannegieter, Kaser Nazir, Susan A. Nancarrow, Alan M. Borthwick

**Affiliations:** ^1^ Frank H Netter MD School of Medicine Quinnipiac University Hamden Connecticut USA; ^2^ Guy's and St Thomas' NHS Trust London UK; ^3^ Allied Health Workforce HealthWork International Brisbane Australia; ^4^ Southern Cross University School of Health and Human Sciences East Lismore Australia; ^5^ Faculty of Environmental and Life Sciences School of Health Sciences University of Southampton Southampton UK

**Keywords:** interprofessional conflict, jurisdictional disputes, orthopaedic surgery, podiatric surgery

## Abstract

Interprofessional conflict has long characterised the relationship between UK podiatric surgeons and orthopaedic surgeons, stemming from overlapping professional boundaries and differing regulatory frameworks. This commentary focuses upon the recent memorandum of understanding (MoU) between the Royal College of Podiatry (RCPod) and the British Orthopaedic Foot and Ankle Society (BOFAS), which aims to address key areas of agreement and disagreement while fostering collaboration. It provides a contextual backdrop by illuminating the historical interprofessional conflict preceding the MoU and highlights the potential of the MoU to enhance patient outcomes, improve workforce sustainability and bridge historical divides. It is clear that achieving lasting progress will require continued dialogue and mutual recognition of each profession's contributions to modern healthcare. These issues also hold broader relevance for interprofessional relations and healthcare policy worldwide.

## 1 Introduction

In November 2024, a memorandum of understanding (MoU) was signed by the Royal College of Podiatry (RCPod) and the British Orthopaedic Foot and Ankle Society (BOFAS), potentially signalling a shift in interprofessional relations, which, historically, have been challenging [1, 2, 3, 4, 5]. Published in full by BOFAS on 15th November 2024 [6], it identifies 8 areas upon which there is agreement while also noting 7 points of continuing disagreement. The aim of this commentary is to review the extent to which the MoU addresses the underlying sources of interprofessional disagreements and to consider from this the prospects for their resolution. In order to do so effectively, a background context is provided which helps explain the underlying historical interprofessional conflict between podiatric and orthopaedic surgery.

Interprofessional conflict has long been recognised as a ‘fundamental fact of professional life’ [7]. It is a dynamic process in which boundaries may, over time, change, sometimes through a negotiated transition, more often through interprofessional conflict [8, 9, 10, 11, 12, 13, 14, 15, 16]. Conflict between professional groups is most acutely observed where overlapping boundaries in core areas of practice occur.

The emergence of invasive foot surgery practiced by nonmedically qualified podiatrists represents one such exemplar, which has led to years of conflict, competition and disagreement, not only in the United Kingdom, but also, as noted very recently, in Australia and the United States of America [2, 3, 4, 5, 17, 18, 19, 20].

A further context is the ongoing government health policy agenda based on greater ‘transdisciplinary’ working in which professional boundaries are no longer rigidly observed but shared across professions where sufficient expertise enables it [8, 9, 21]. The UK government health policy agenda promotes workforce flexibility as a means to solve the growing crisis in workforce capacity, with a shrinking pool of younger people entering the professions and an increasing number of ageing baby‐boomers retiring and in need of healthcare [21, 22, 23, 24, 25].

The traditional model of professionalism is based upon both monopoly and autonomy, and is largely centred, in healthcare, around a traditional hierarchy led by medicine [11, 12, 13, 14, 15, 26]. Neoliberal policies, particularly evident since the 1980s, have steadily sought to challenge these exclusive, exclusionary ways of working, given the need to resolve workforce shortages [7, 12].

It appears, therefore, that health policy objectives which recognise a need for flexible, innovative ways of working are at odds with the exclusionary tactics adopted by most professions when defending their established roles and task domains, particularly given the demographic trend is set to continue for some time [10, 22, 27, 28].

Within that context, podiatric surgery in the United Kingdom, United States of America and Australia has become a legitimate, mainstream provider of foot surgery, firmly regulated and legally recognised [1, 3, 20]. In the United KIngdom, it has become integrated within the National Health Service as well as the private sector, with senior podiatric surgeons appointed to consultant grade since the 1990s [1].

## 2 The Memorandum of Understanding: A Step Forward?

The MoU marks the first joint communications issued by the RCPod and BOFAS and thus represents a milestone in what has been a complex and troubled relationship. Although there remain key areas of disagreement, there is a broad consensus that sustainable foot surgery provision in the United Kingdom is essential to meet the needs of patients and the broader policy agenda.

Although the areas of relative agreement may reflect many of those issues that do not tend to provoke jurisdictional disputes, it is clear that some real progress has been made in certain areas of concern [7]. For example, agreement that patients and the public would benefit from closer working practices remains uncontentious and reflects a common professional rhetoric often used to stress the altruistic nature of healthcare professionalism [7, 11]. An opportunity to work together on a national registry of foot and ankle operations, and to collaborate on research, indicates progress. There is even tacit agreement that there may be ‘potential’ joint training opportunities to be explored—but only ‘given equivalent regulation’ (11.8, p10), qualifying a particular concern by BOFAS that there should be ‘more robust’ Health and Care Professions Council (HCPC) regulation (MoU, 12.3, p11).

Perhaps, some real change is discernible in the inclusion within the MoU of the Health and Care Professions Council annotation requirements, outlined in detail in the appendices, which specify 19 standards to be continually met by podiatrists practising podiatric surgery as well as 15 standards of proficiency, 10 standards of conduct, performance and ethics, 5 standards of continuing professional development and prescribing standards, all of which provide a clear example of the key developments in regulation and governance adopted as part of the annotation process, fully enacted since 2020.

Indeed, as part of the process, the HCPC ‘held two meetings to bring together key stakeholders with an interest in podiatric surgery, including the College of Podiatry, NHS Education for Scotland (NES), the British Orthopaedic Foot and Ankle Society (BOFAS), the British Orthopaedic Association (BOA), the Royal College of Surgeons (RCS) and the General Medical Council (GMC). We took account of their comments in preparing these standards for consultation’ (1.10, p6) [29]. Given the responses in the public consultation, the HCPC regarded annotation as a proportionate response, following the Right Touch Regulation principles established by the Professional Standards Authority (PSA). It is perhaps worth clarifying that the concept of ‘right touch regulation’ has been long established by the Better Regulation Executive and widely deployed by the Professional Standards Authority, and is not, as is commonly (and perhaps mischievously) misunderstood, ‘light touch’ regulation. The PSA oversees UK healthcare regulation, including that of the HCPC and General Medical Council (GMC), in line with the Better Regulation Executive's principles [30].

Furthermore, the MoU includes reference to podiatric surgeons as ‘independent, autonomous surgeons who are able to undertake a range of surgical techniques within the foot and its associated structures’, the ‘vast majority’ of whom have become independent prescribers since legislation was laid in 2013 (MoU, item 5.17 and 5.18). Item 5.19 of the MoU also states that ‘podiatric surgeons can optimise patients for surgery, admit them for surgery and manage post‐op complications surgically and pharmacologically. This is done in a framework of governance and multidisciplinary Team (MDT) working’, acknowledging the contribution of podiatric surgeons to healthcare.

In the MoU's summary of disagreements, those areas still contested are brought into sharp relief. Although many have persisted over nearly 50 years, in practice, transdisciplinary role changes have gathered momentum, in line with the broader government health policy agenda [21]. Greater regulation and governance of podiatric surgery have followed as part of that agenda, notably with HCPC annotation and its raft of requirements.

Nevertheless, one key concern for BOFAS remains the desire to ensure the processes set out by the GMC should apply to all those practising surgery. It maintains that the training and regulation of podiatric surgeons are ‘not sufficient’ to ensure the safe treatment of patients (MoU, 12.3, p11). This implies a desire to ensure medical oversight of podiatric surgery, exclusively through the GMC.

Interestingly, concerns about the sufficiency of podiatric surgery training and education echoes similar concerns expressed very recently by the Royal Australian College of Surgeons in the public consultation to the review of podiatric surgery in Australia [20]. The review's final report concluded that ‘…concerns about the quality of education and training of podiatric surgeons are not supported by the evidence’ (P.4). Certainly, the overall duration of training and education, as noted in the MoU, varies between each group, 11 years as a podiatric surgeon and 15 years as an orthopaedic surgeon. The difference in length of training almost certainly reflects the point raised by the HCPC, that it ‘…recognise[s] that there may be differences in arrangements. Orthopaedic surgeons gain a wide range of experience before deciding to sub‐specialise in the foot. Podiatrists practising podiatric surgery focus solely on surgical procedures of the foot and associated structures’ (5.10, p20) [29]. This is illustrated by the fact that trauma and orthopaedic surgeons are required to complete a combined minimum of 20 elective osteotomy procedures from the following structures: 1st metatarsal, proximal tibia, distal femur, hip, humerus, wrist, hand, paediatric and spinal, to gain their certificate of completion of specialist training, whereas podiatric surgeons must complete 850 foot procedures [31, 32].

In the past, orthopaedic views on the training and education of podiatric surgeons, as evident in the literature and media, have often expressed similar views to that in the MoU [4]. Indeed, a recent editorial, published shortly before the MoU was signed, presented orthopaedic concerns framed in a narrative that devalued the validity of training and regulation of podiatric surgeons in the United Kingdom and the United States of America, implying a lack of adequate governance [33]. Although the article acknowledged the need for ‘clinicians with different backgrounds and training to work closely together…’, it suggested that nonmedically trained surgeons may have questionable capabilities [33]. At its core is the view that such close, collaborative work should be integrated under the direct oversight of the General Medical Council, ensuring podiatric surgeons are subject to medical control.

Yet, BOFAS previously ‘recognised the training and skills of podiatrists and operative podiatrists…’ [19]. In 2010, the President of the British Orthopaedic Association remarked that it was ‘undeniable that this is a group whose skills can offer genuine benefit for patients’ [34]. Similar views have been expressed periodically across the decades [35, 36, 37]. Equally, government endorsement of podiatric surgery has been evident for years, notably expressed by Lord Hunt of King's Heath, as ‘the development of podiatric surgery is consistent with the Government's desire to end traditional demarcations which may inhibit the modernisation of services’ [38].

Use of the title ‘podiatric surgeon’ has long been a bone of contention, with repeated claims that its use is ‘misleading’ and that it should be replaced by alternative titles, such as ‘operative podiatrist’, ‘podiatric proceduralist’ or ‘podiatric technician’ [20, 33, 39, 40, 41, 42, 43]. In the MoU, BOFAS recommends the term presently adopted in the HCPC annotation (podiatrist practising podiatric surgery), however recognising that, as was noted at the 2023 BOFAS Annual Congress, ‘arguments based on title will fail’ [44].

The RCPod has consistently provided advice, documentation and patient literature for over a decade, clarifying that there is no intent to portray podiatric surgeons as registered medical professionals. The title ‘surgeon’, as the RCPod has emphasised, must always be prefixed with ‘podiatric’. Furthermore, the MoU explicitly discourages orthopaedic surgeons from adopting the title ‘podiatric surgeon’.

## 3 Conclusion

Does the recent memorandum of understanding offer hope that these complex issues might be resolved? Realistically, there remains a gulf between them on a small number of key issues, grounded in a degree of historical protectionism. However, overall, the MoU suggests there are sufficient grounds for genuine optimism.

What is likely to be important for the future are improved services for patients, maximising workforce capacity, promoting transdisciplinary and interprofessional working and ensuring healthcare sustainability. It is these issues with which the RCPod and BOFAS must grapple, and the MoU may yet signal an important step forward in that direction.

## Author Contributions

**Ewan Kannegieter:** conceptualization, data curation, writing – original draft, writing – review and editing. **Kaser Nazir:** conceptualization, writing – review and editing. **Susan A. Nancarrow:** conceptualization, data curation, formal analysis, writing – original draft, writing – review and editing. **Alan M. Borthwick:** conceptualization, data curation,formal analysis, writing – original draft, writing – review and editing.

## Ethics Statement

The authors have nothing to report.

## Consent

The authors have nothing to report.

## Conflicts of Interest

E.K. is the dean of the Faculty of Podiatric Surgery, Royal College of Podiatry and was involved in negotiating the MoU. K.N. is a member of the Faculty Board of Podiatric Surgery, Royal College of Podiatry.

## Data Availability

The authors have nothing to report.
